# Roles of estradiol levels on the day of human chorionic gonadotrophin administration in the live birth of patients with frozen embryo transfer

**DOI:** 10.1002/jcla.23422

**Published:** 2020-07-26

**Authors:** Haixiao Chen, Jiali Cai, Lanlan Liu, Xiaohua Sun

**Affiliations:** ^1^ Reproductive Medicine Center Chenggong Hospital Affiliated to Xiamen University Xiamen China

**Keywords:** estradiol, frozen embryo transfer, human chorionic gonadotrophin, live‐birth rates, predictor

## Abstract

**Background:**

Estradiol (E_2_) is an important hormone in women. Changes of serum E_2_ levels may affect the endometrial receptivity for embryo implantation and thus affect pregnancy outcomes. This study was to assess the association between serum E_2_ levels on the day of human chorionic gonadotrophin (HCG) administration and live‐birth rates in patients with frozen embryo transfer (FET).

**Methods:**

Totally 2071 women receiving long protocols of long‐acting gonadotropin‐releasing hormone (GnRH) agonists were enrolled. According to the E_2_ levels on the day of HCG administration, these patients were divided into four groups: 676 cases of E_2_ ≤ 3051 pg/mL in Q_1_ group, 676 cases of 3051 pg/mL < E_2_ ≤ 4558 pg/mL in Q_2_ group, 675 cases of 4558 pg/mL < E_2_ ≤ 6718 pg/mL in Q_3_ group, and 674 cases of E_2_ > 6718 pg/mL in Q_4_ group. The clinical indicators including female age, body mass index (BMI), duration of infertility, infertility styles, treatment protocols, hormone levels, total antral follicle count, endometrial thickness, top‐level embryos, and live‐birth rates were analyzed, and multivariable logistic model was conducted to select significant variables.

**Results:**

Significant differences were observed for the female age (OR = 0.965, 95% CI: 0.946‐0.985, *P* < .001), total antral follicle counts (OR = 1.025, 95% CI: 1.008‐1.043, *P* = .004), transferring what day of embryos (OR = 1.242, 95% CI: 1.137‐1.356, *P* < .001), endometrial thickness (OR = 1.058, 95% CI: 1.004‐1.115, *P* = .035), top‐level embryos (OR = 1.416, 95% CI: 1.157‐1.731, *P* = .001), and E_2_ levels on HCG day >6781 pg/mL (OR = 1.344, 95% CI: 1.069‐1.690, *P* = .011) between live‐birth and non‐live‐birth groups. The area under the curve (AUC) for E_2_ levels on HCG day was 0.558, the sensitivity was 54.75%, and the specificity was 55.10%.

**Conclusion:**

Serum E_2_ level on HCG day was an independent predictor of live‐birth achievement in patients with FET.

## 1 INTRODUCTION

Infertility is a highly prevalent problem of global proportions which is estimated to affect as many as 186 million people worldwide. Although more than half of the world's childless cases are caused by male infertility, the social burden is still on women. The infertility rate of the population at childbearing age is up to 15%‐20%, and over 20 million couples have fertility issues in China.^1^ Assisted reproductive techniques (ARTs), as an emerging treatment for infertility, are achieved more attention in recent decades. Since the birth of the first test‐tube baby in 1978,^2^ in vitro fertilization and embryo transfer (IVF‐ET) is gradually applied in clinic. The previous study revealed that the successful pregnancy rates after IVF‐ET were 39% in America.^3^ Frozen embryo transfer (FET) is an assisted reproductive technology procedure in which can reduce the risk of ovarian hyperstimulation syndrome (OHSS), maximize the utilization of embryo in oocyte retrieval per cycle,^4^ and provide a choice for patients without conducting the fresh embryo transfer. Evidences showed that FET can obtain better outcomes in perinatal period and live birth, such as ectopic pregnancy, premature birth, low birth weight neonates, and placenta previa.^5^, ^6^, ^7^

Implantation is a complex process regulating by multiple factors of which human chorionic gonadotrophin (HCG) is one of the most important factors.^8^ HCG, a glycoprotein, serves as a surrogate for luteinizing hormone (LH) used to induce the maturation and ovulation of oocytes in ovarian stimulation cycles,^9^ which is an early embryonic signal secreted via the embryo before implantation in primates. HCG can stimulate the connective tissue of follicle softened to easily separate the oocyte cumulus complex from the follicle wall, and enable to perform the aspiration during oocyte retrieval. Estradiol (E_2_), one of the three main self‐produce estrogens, is an important hormone in women. E_2_ has a crucial influence on the growth and function of the female reproductive system and the mammary gland in physiological conditions. Evidences showed that the serum E_2_ level is an integral part of evaluating response to controlled ovarian stimulation, and the elevated E_2_ levels may affect the endometrial receptivity for embryo implantation owing to controlled ovarian hyperstimulation.^10^

In recent decades, the association between serum E_2_ levels on the day of HCG administration on pregnancy outcomes in IVF was assessed.^11^, ^12^ To the best our knowledge, however, the influence of E_2_ levels on live‐birth rates in embryo implantation has been rarely reported. Herein, we investigated the effect of E_2_ levels on the day of HCG administration on live‐birth rates in patients with FET.

## 2 METHODS

### 2.1 Patients

A total of 2071 patients with FET admitted to Chenggong Hospital Affiliated to Xiamen University were enrolled from 2012 to 2017 in this study. According to the E_2_ levels on the day of HCG administration, these patients were divided into four groups, containing 676 cases of E_2_ ≤ 3051 pg/mL in Q_1_ group, 676 cases of 3051 pg/mL < E_2_ ≤ 4558 pg/mL in Q_2_ group, 675 cases of 4558 pg/mL < E_2_ ≤ 6718 pg/mL in Q_3_ group, and 674 cases of E_2_ > 6718 pg/mL in Q_4_ group. A number of baseline clinical indicators, including female age, body mass index (BMI), duration of infertility, infertility styles, treatment protocols, hormone levels, total antral follicle count, endometrial thickness, top‐level embryos, and live‐birth rates were noted. Endometrial thickness was detected using B‐scan ultrasonography. This study was approved by the Institutional Review Board (IRB) of Chenggong Hospital Affiliated to Xiamen University.

### 2.2 Inclusion and exclusion criteria

Patients who met the inclusion criteria were included: (a) ≥18 years old; (b) all received the long protocols of long‐acting gonadotropin‐releasing hormone (GnRH) agonists with one of the treatments, including in vitro fertilization (IVF), intracytoplasmic sperm injection (ICSI), or rescue ICSI (RICSI); and (c) without taking drugs or excessive drinking.

The exclusion criteria were as follows: (a) patients with endometriosis or polycystic ovary syndrome (PCOS); (b) merged with ovary malignant tumor; (c) incorporated with cardiovascular, digestive, urinary, blood systems or other diseases; (d) a history of repeated implantation failure (RIF); and (e) congenital dysplasia without uterus or immature uterus cannot be pregnancy.

### 2.3 Scoring criteria for embryo selection

On day 3 of cleavage stage, the scoring criteria for embryo selection were based on the previous study.^13^ The cell number of top‐level embryos was 7, 8, and 9, respectively. In the merged period (M), the cells presented the uniform size, regular shape, intact zona pellucida, uniform and clear cytoplasms, with no particles, and the debris ranged 0%‐5%.

The blastular scoring on day 5 was on the basis of the Gardner score.^14^ The top‐level blastocysts were defined as fully expanded blastocysts, namely the blastular cavity was full of embryos with larger volume and thinner zona pellucida, or the hatching blastocysts part of which were escaped from the zona pellucida. The number of cells in the inner cell group was large and closely arranged, the upper cells of the trophoblast were composed of more cells, and the structure was tight.

### 2.4 Ovarian stimulation

The ovarian stimulation was conducted using the long protocols of long‐acting GnRH agonists in this study.^15^ In the luteal (on day 7 after ovulation) or follicular phases (on day 1 or 2 during menstruation), 1.25‐3.75 mg of the long‐acting GnRH agonists (Diphereline, Tianjin Yipusheng Pharmaceutical Co., Ltd.) was intramuscularly injected. The levels of serum E_2_, follicle‐stimulating hormone (FSH), LH, and vaginal ultrasound were detected after 14‐28 days. After hormone levels were up to the down‐regulation standard (E_2_ < 50 pg/mL, FSH and LH < 5 IU/mL, endometrial thickness <5 mm), human menopausal gonadotrophin (HMG) (Zhuhai Lizhu Pharmaceutical Co., Ltd.) and/or recombinant human FSH (Gonal‐f, Serono Pharmaceutical Co., Ltd.) were injected at 112.5‐225 IU/d. According to the development of follicles and the levels of serum sex hormone, the HCG trigger was added at the appropriate time.

### 2.5 Embryo transfer

#### 2.5.1 Natural cycle

According to the previous monitoring of ovulation, the vaginal ultrasound was used to monitor the development of follicles on day 9‐11 of menstruation, which was suitable for patients with regular ovulation of menstruation. When the diameter of the follicle was about 16 mm, the urine LH was monitored and the E_2_, LH, and P were detected by blood drawing until the day of ovulation. On the day of ovulation, the patients took 20 mg of dydrogesterone (Abbott), twice daily. On day 3 after ovulation, the embryos in the cleavage stage were thawed and transplanted. The blastocysts were thawed and transplanted on day 5 after ovulation.

#### 2.5.2 Hormone replace treatment (HRT)

On the second day of menstruation, the patients underwent B‐scan ultrasonography, which showed no abnormalities in the endometrium, so the interference factors including ovarian cysts, larger follicles, were excluded. The patients orally took 6‐8 mg of progynova (Bayer), and the dosage was appropriately adjusted when the endometrial growth was monitored by B‐scan ultrasonography after 8‐10 days. On day 14‐16, when the endometrial thickness was ≥8 mm, 40 mg/d of progesterone (Xianju) was intramuscularly injected, then dydrogesterone (Abbott) was orally taken 20 mg/bid the next day. The embryos in cleavage stage were performed the FET on day 5 of progesterone injection. The blastocysts were conducted the frozen blastocyst transfer on day 7 of progesterone injection. After transplantation, the original dose of hormone replacement and luteum support were continued.

### 2.6 Serum hormone measurements

Serum hormone measurements were carried out using chemoluminescence immunoassays (ECLIA, UniCel DxI 800, Beckman Coulter). The levels of these hormones including basal FSH, basal LH, basal prolactin (PRL), basal E_2_, basal testosterone (T), basal progesterone (P), and basal gonadotropin (Gn) were detected in this study.

### 2.7 Statistical analysis

Statistical analysis was performed using SPSS 24.0 (SPSS, Inc). Continuous data were presented as the median (P_25_, P_75_) and analyzed by Kruskal‐Wallis test. Categorical data were presented as n and analyzed using chi‐squared test. One‐way analysis of variance (ANOVA) with least significant difference post hoc test was utilized to compare the groups. Multivariable logistic regression analysis was used to identify the variables that may contribute to the live‐birth rate. The predictive value of E_2_ day HCG on live‐birth rates was assessed by receiver operating characteristic (ROC) curve analysis. *P* < .05 was considered to indicate a statistically significant difference.

## 3 RESULTS

### 3.1 The characteristics of patients with FET

A total of 2071 FET patients receiving the long protocols of long‐acting GnRH agonists were enrolled in this study. The characteristics of patients were shown in Table 1. There were significant differences in age, BMI, duration of infertility, treatments (IVF, ICSI, and RICISI), basal FSH, basal LH, basal T, total Gn, total antral follicle counts, transferring what day of embryos, top‐level embryos, and live‐birth rates among the four groups (*P* < .05).

**TABLE 1:** The characteristics of patients with FET

Variables	Q_1_ (n = 676)	Q_2_ (n = 676)	Q_3_ (n = 675)	Q_4_ (n = 674)	*P*
Age, y, median (P_25_, P_75_)	30 (28, 33)	30 (27, 33)	29 (27, 31)^a^, ^b^	29 (27, 32)^a^, ^b^	.001
BMI, kg/m^2^, median (P_25_, P_75_)	21.4 (19.6, 23.3)	21 (19.2, 22.5)^a^	20.3 (18.8, 22.0)^a^, ^b^	19.8 (18.6, 21.325)^a^, ^b^, ^c^	.001
Duration of infertility, y, median (P_25_, P_75_)	3.5 (2, 6)	3.2 (2, 6)	3 (2, 5)	3 (2,5)	.027
Infertility styles, n (%)					
Secondary infertility	363 (53.9)	343 (51.0)	328 (48.7)	326 (48.4)	.152
Primary infertility	310 (46.1)	330 (49.0)	346 (51.3)	347 (51.6)	
Treatments, n (%)					
IVF	488 (72.2)	513 (75.9)	473 (70.1)	503 (74.6)	.045
ICSI	142 (21.0)	130 (19.2)	169 (25.0)	144 (21.4)	
RICSI	46 (6.8)	33 (4.9)	33 (4.9)	27 (4.0)	
Basal FSH, mIU/L, median (P_25_, P_75_)	6.73 (5.78, 8.02)	6.48 (5.58, 7.52)^a^	6.44 (5.58, 7.38)^a^	6.37 (5.50, 7.34)^a^	.001
Basal LH, mIU/L, median (P_25_, P_75_)	4.1 (3.08, 5.63)	4.36 (3.44, 5.82)^a^	4.79 (3.52, 6.27)^a^, ^b^	4.84 (3.80, 6.06)^a^, ^b^	.001
Basal PRL, ng/mL, median (P_25_, P_75_)	13.54 (9.86, 19.48)	14.28 (10.49, 20.02)	14.59 (10.53, 20.16)	14.97 (10.95, 20.22)	.051
Basal E_2_, pg/mL, median (P_25_, P_75_)	38 (28, 51)	39 (29.2, 51)	39 (29,5 3)	39 (30, 56)	.174
Basal T, ng/L, median (P_25_, P_75_)	0.37 (0.27, 0.48)	0.38 (0.28, 0.52)	0.38 (0.29, 0.53)	0.39 (0.30, 0.52)^a^	.013
Basal P, ng/L, median (P_25_, P_75_)	0.65 (0.42, 0.99)	0.68 (0.44, 0.96)	0.69 (0.46, 1.09)	0.68 (0.45, 0.99)	.144
Total Gn, IU, median (P_25_, P_75_)	2400 (2025, 2700)	2250 (1837, 2700)^a^	2250 (1800, 2588)^a^	2250 (1866, 2625)	.001
Total antral follicle counts, n, median (P_25_, P_75_)	10 (8, 13)	11 (8, 15)^a^	12 (9, 15)^a^, ^b^	11 (9, 15)^a^	.001
Transferring what day of embryos, median (P_25_, P_75_)	5 (3, 5)	5 (5, 5)^a^	5 (5, 5)^a^	5 (5, 5)^a^, ^b^	.001
Embryos transferred, n, median (P_25_, P_75_)	2 (1,2)	2 (1,2)	2 (1, 2)	2 (1, 2)	.848
Endometrial thickness, mm, median (P_25_, P_75_)	8.40 (7.60, 9.40)	8.35 (7.60, 9.40)	8.30 (7.70, 9.20)	8.40 (7.80, 9.30)	.889
Top‐level embryos, %	11.2	18.5^a^	21.3^a^	20.6^a^	.001
Live‐birth rates, %	37.9	42.0	46.5^a^	51.3^a^, ^b^	.001

Note

Q_1_: E_2_ levels on HCG day ≤ 3051 pg/mL; Q_2_: 3051 pg/mL < E_2_ levels on HCG day ≤ 4558 pg/mL; Q3: 4558 pg/mL < E_2_ levels on HCG ≤ 6781 pg/mL; Q_4_: E_2_ levels on HCG day > 6781 pg/mL.

Abbreviations: BMI, body mass index; E_2_, estradiol; FSH, follicle‐stimulating hormone; Gn, gonadotropin; HCG, human chorionic gonadotrophin; ICSI, intracytoplasmic sperm injection; IVF, in vitro fertilization; LH, luteinizing hormone; P, progesterone; PRL, prolactin; RICSI, rescue ICSI; T, testosterone.

^a^ Compared with Q_1_, *P* < .05.

^b^ Compared with Q_2_, *P* < .05.

^c^ Compared with Q_3_, *P* < .05.

### 3.2 Single‐factor analysis of live‐birth rates

Based on the pregnancy outcomes, 2071 cases were divided into the live‐birth group (n = 1501) and no‐live‐birth group (n = 1200), respectively. The results of the single‐factor analysis showed that the statistical differences were prominent in female age (*χ* ^2^ = 4.240, *P* = .001), duration of infertility (*χ* ^2^ = 2.451, *P* = .014), basal FSH (*χ* ^2^ = 2.875, *P* = .004), basal T (*χ* ^2^ = 3.046, *P* = .002), total Gn (*χ* ^2^ = 3.419, *P* = .001), total antral follicle counts (*χ* ^2^ = 3.701, *P* = .001), transferring what day of embryos (*χ* ^2^ = 5.229, *P* = .001), endometrial thickness (*χ* ^2^ = −2.471, *P* = .013), top‐level embryos (*χ* ^2^ = 20.617, *P* = .001), and E_2_ levels on HCG day (*χ* ^2^ = 5.210, *P* = .001) between the live‐birth and no‐live‐birth groups (Table 2).

**TABLE 2:** Single‐factor analysis of live‐birth rates

Variables	Non‐live birth (n = 1501)	Live birth (n = 1200)	*χ* ^2^	*P*
Age/female, y, median (P_25_, P_75_)	30 (27, 33)	29 (27, 32)	4.240	.001
BMI, kg/m^2^, median (P_25_, P_75_)	20.6 (19.1, 22.2)	20.5 (19, 22.3)	0.140	.889
Duration of infertility, y, median (P_25_, P_75_)	3.3 (2, 6)	3 (2, 5)	2.451	.014
Infertility styles, n (%)				
Secondary infertility	769 (51.05)	591 (49.30)	1.356	.244
Primary infertility	724 (48.50)	609 (50.70)		
Treatment, n (%)				
IVF	1098 (73.20)	879 (73.30)	2.102	.350
ICSI	318 (21.20)	267 (22.30)		
RICSI	85 (5.70)	54 (4.50)		
Basal FSH, mIU/L, median (P_25_, P_75_)	6.59 (5.63, 7.66)	6.39 (5.57, 7.41)	2.875	.004
Basal LH, mIU/L, median (P_25_, P_75_)	4.51 (3.42, 5.86)	4.64 (3.51, 6.11)	1.687	.092
Basal PRL, ng/mL, median (P_25_, P_75_)	14.18 (10.25, 19.82)	14.53 (10.60, 20.23)	1.742	.081
Basal E_2_, pg/mL, median (P_25_, P_75_)	38 (29, 53)	40 (29, 52)	0.839	.401
Basal T, ng/L, median (P_25_, P_75_)	0.37 (0.28, 0.5)	0.39 (0.30, 0.52)	3.046	.002
Basal P, ng/L, median (P_25_, P_75_)	0.69 (0.44, 1.01)	0.66 (0.44, 1.01)	0.836	.403
Total Gn, IU, median (P_25_, P_75_)	2287.5 (1875, 2700)	2250 (1800, 2587.5)	3.419	.001
Total antral follicle counts, n, median (P_25_, P_75_)	11 (8, 14)	11.5 (9, 15)	3.701	.001
Transferring what day of embryos, median (P_25_, P_75_)	5 (3, 5)	5 (5, 5)	5.229	.001
Embryos transferred, n, median (P_25_, P_75_)	2 (1, 2)	2 (1, 2)	0.156	.876
Endometrial thickness, mm, median (P_25_, P_75_)	8.30 (7.60, 9.20)	8.40 (7.80, 9.40)	−2.471	.013
Top‐level embryos, %	14.90	21.70	20.617	.001
E_2_ levels on HCG day, pg/mL, median (P_25_, P_75_)	4411 (2878, 6290)	4814 (3311, 7115)	5.210	.001

Abbreviations: BMI, body mass index; E_2_, estradiol; FSH, follicle‐stimulating hormone; Gn, gonadotropin; HCG, human chorionic gonadotrophin; ICSI, intracytoplasmic sperm injection; IVF, in vitro fertilization; LH, luteinizing hormone; P, progesterone; PRL, prolactin; RICSI, rescue ICSI; T, testosterone.

### 3.3 Multivariable logistic analysis of live‐birth rates

**FIGURE 1:**
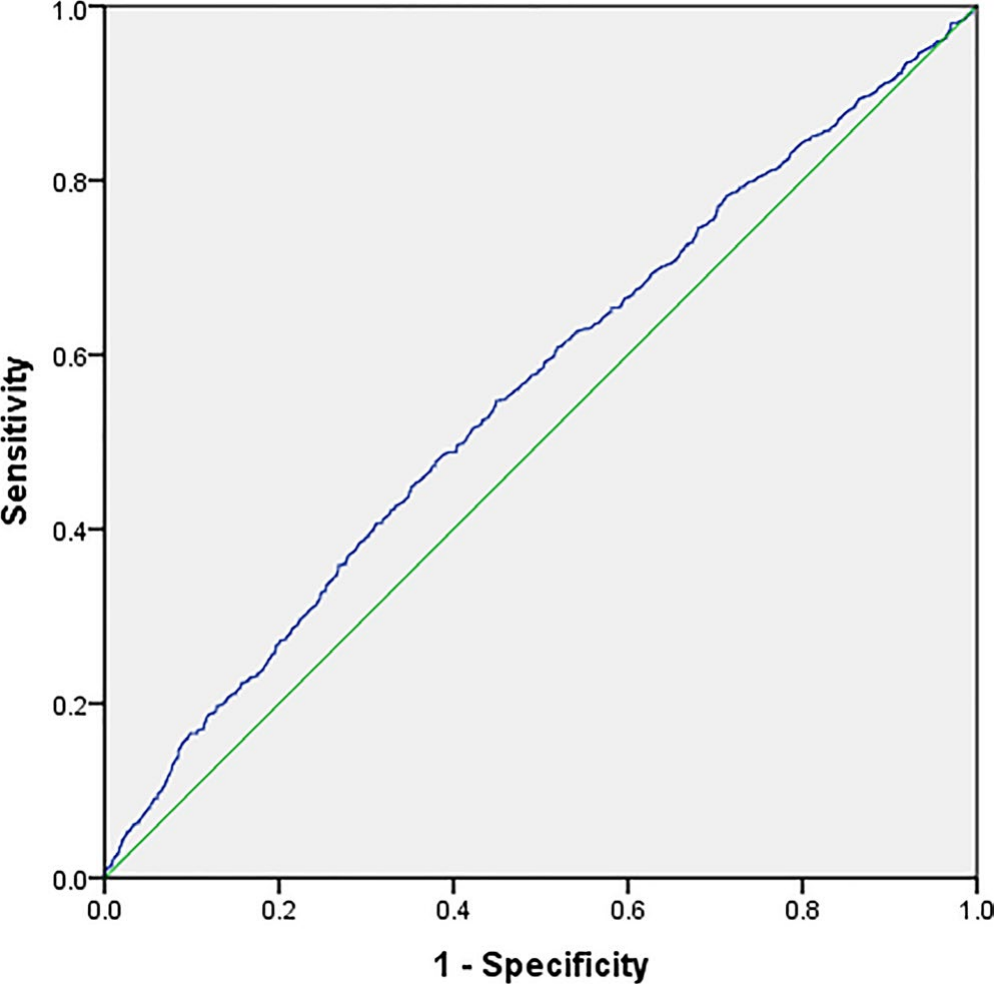
The ROC curve of serum E_2_ level on the day of HCG administration on live‐birth rates in FET patients

The live‐birth rate as the dependent variable, and the independent variables including female age, total antral follicle counts, transferring what day of embryos, endometrial thickness, top‐level embryos, and E_2_ levels on HCG day were analyzed (Table 3). Significant effects on the live birth were observed for the female age (OR = 0.965, 95% CI: 0.946‐0.985, *P* < .001), total antral follicle counts (OR = 1.025, 95% CI: 1.008‐1.043, *P* = .004), transferring what day of embryos (OR = 1.242, 95% CI: 1.137‐1.356, *P* < .001), endometrial thickness (OR = 1.058, 95% CI: 1.004‐1.115, *P* = .035), top‐level embryos (OR = 1.416, 95% CI: 1.157‐1.731, *P* = .001), and E_2_ levels on HCG day >6781 pg/mL (OR = 1.344, 95% CI: 1.069‐1.690, *P* = .011). To assess the predictive value of serum E_2_ level on the day of HCG administration on live‐birth rates, a ROC curve was exhibited in Figure 1. The area under the curve (AUC) of serum E_2_ level on HCG day was 0.558, and the Youden's index was 0.0985. The cutoff value maximizing the composite of specificity and sensitivity in the prediction of the live‐birth rate was 4599. According to the ROC curves, the sensitivity and specificity were 54.75% and 55.10%, respectively.

**TABLE 3:** Multivariable logistic analysis of live‐birth rates

Variables	*β*	SE	Wald	*P*	OR	95% CI	
						Lower	Upper
Constant	−1.121	0.63	5.851	.016	‐	‐	‐
Age/female	−0.036	0.010	12.172	<.001	0.965	0.946	0.985
Total antral follicle counts	0.025	0.009	8.075	.004	1.025	1.008	1.043
Transferring what day of embryos	0.216	0.045	23.010	<.001	1.242	1.137	1.356
Endometrial thickness	0.056	0.027	4.450	.035	1.058	1.004	1.115
Top‐level embryos (yes)	0.348	0.103	11.435	.001	1.416	1.157	1.731
Q_2_	−0.004	0.115	0.001	.972	0.996	0.794	1.250
Q_3_	0.110	0.117	0.888	.527	1.077	0.856	1.355
Q_4_	0.296	0.117	6.405	.011	1.344	1.069	1.690

Note

Q_1_: E_2_ levels on HCG day ≤ 3051 pg/mL; Q_2_: 3051 pg/mL < E_2_ levels on HCG day ≤ 4558 pg/mL; Q3: 4558 pg/mL < E_2_ levels on HCG ≤ 6781 pg/mL; Q_4_: E_2_ levels on HCG day > 6781 pg/mL.

Abbreviations: E_2_, estradiol; HCG, human chorionic gonadotrophin.

## 4 DISCUSSION

Previous studies discovered that high serum E_2_ level may be not in favor of embryo implantation^16^; nevertheless, effects of serum E_2_ levels on live‐birth rates have been not conclusive during the FET cycle. In this present study, we evaluated the effect of serum E_2_ levels on the day of HCG administration on live‐birth rates in patients with FET. Our findings revealed that the total antral follicle counts, transferring what day of embryos, endometrial thickness, and top‐level embryos were risk factors, and the female age was a protective factor of the live birth in FET cycle. Additionally, serum E_2_ level on HCG day was an independent predictor of live‐birth achievement.

In vitro fertilization was performed using the fresh embryos transfer in the early years.^17^ Frozen embryo was conducted when fresh embryo transfer was unsuccessful. A study of 2157 women in a multicenter randomized trial demonstrated that the risk of moderate or severe ovarian hyperstimulation syndrome was lower among patients who underwent the FET in comparison with fresh embryo transfer.^18^ Evidences suggested that embryos were frozen in a fresh IVF cycle, then were thawed and transferred into uterus in the proper condition might improve the pregnancy rates.^19^, ^20^, ^21^ Furthermore, the occurrence of pregnancies after FET can lead to fewer complications such as lower rates of antepartum hemorrhage, and better neonatal outcomes including higher birth weight and lower risk of perinatal death.^22^ A great number of FET are applied for infecund individuals in various countries,^23^, ^24^, ^25^ which overcome the deficiencies of fresh embryo transfer and provide more choices for clinicians and patients.

The successful rate of implantation is influenced by endometrial receptivity and the synchronization between embryos and endometria to some extent.^26^ Early studies founded that E_2_ was essential for the receptive establishment^27^ and endometrial proliferation, and played a determinant role in implantation, decidualization, and early embryo development.^28^ The concentration dependence of serum E_2_ levels on pregnancy rates has been mentioned.^29^ Previous studies reported the prognostic value of E_2_ level at the time of HCG triggering during the fresh IVF/ICSI cycles, which may have an impact on the number of oocytes and top‐level embryos.^30^ The finding we discovered that the top‐level embryo was a risk factor for the live birth, and as the increased number of the top‐level embryos, the live‐birth rates were increased. In addition, the endometrial thickness in the live‐birth group was higher than that in the non‐live‐birth group. Previous studies showed that increased endometrial thickness can significantly improve pregnancy outcomes among women undergoing IVF‐ET.^31^, ^32^ On the day of HCG administration or embryo transfer, the endometrium pattern and thickness were obviously associated with the rates of clinical pregnancy and embryo implantation.^33^ Our results founded that serum E_2_ level on the day of HCG administration was an independent predictor of live‐birth achievement, and high serum E_2_ level on HCG day was associated with decreased live‐birth rates in patients with FET. The impaired oocyte maturation and the decreased pregnancy achievement caused by elevated E_2_ levels have been mentioned.^34^, ^35^ Clinical studies suggested that high E_2_ levels could adversely affect perinatal outcomes. The occurrence of complications associated with abnormal placenta was significantly increased when serum E_2_ level was at >10 000 pmol/L.^36^

Previous studies showed that the alterations of these hormones such as basal LH, basal PRL, and basal P levels were associated with pregnancy in embryo transfer.^37^, ^38^, ^39^ In our study, there were no statistical differences in the levels of basal LH, basal PRL, and basal P between the live‐birth group and the no‐live‐birth group. Then, the significant variables with single‐factor analysis were further analyzed using stepwise multivariable logistic analysis. No differences in the levels of basal FSH, basal LH, basal T, and total Gn were discovered between the two groups. It was indicated that the results may be related to the individual physical condition, age, etc.

The superiority of this study was that few previous researches had investigated the association between serum E_2_ level on HCG day and the live‐birth rates in patients who undergoing FET, especially in Chinese population. It was the fact that serum E_2_ levels on HCG day may be an effective predictor of live‐birth achievement in patients with FET, which was beneficial for clinicians to effectively control the E_2_ levels to improve the live‐birth rates. There was a limitation that should be warranted caution for interpreting the data in this study. Our investigation was a retrospective study based on a single center, which included only 2071 infertile women. Thus, prospective multicenter studies with larger samples should be needed for further verification of the effect of serum E_2_ level on HCG day on live‐birth rates in clinic.

## 5 CONCLUSION

In summary, the total antral follicle counts, transferring what day of embryos, endometrial thickness, and top‐level embryos were risk factors, and the female age was a protective factor of the clinical live birth in FET cycle. Our findings demonstrated that high serum E_2_ level on HCG day was associated with decreased live‐birth rates in patients with FET.

## AUTHOR CONTRIBUTIONS

All authors read and approved the final manuscript. HXC designed the study, wrote and critically reviewed the manuscript. JLC and LLL involved in the data curation, validation and formal analysis. XHS involved in supervision and validation.

## ETHICAL APPROVAL

This study was approved by the Institutional Review Board (IRB) of Chenggong Hospital Affiliated to Xiamen University and had been performed in accordance with the ethical standards laid down in the 1964 Declaration of Helsinki and its later amendments.

## INFORMED CONSENT

Informed consent was obtained from all individual participants included in the study.
